# Microclimate variables of the ambient environment deliver the actual estimates of the extrinsic incubation period of *Plasmodium vivax* and *Plasmodium falciparum*: a study from a malaria-endemic urban setting, Chennai in India

**DOI:** 10.1186/s12936-018-2342-1

**Published:** 2018-05-16

**Authors:** Shalu Thomas, Sangamithra Ravishankaran, N. A. Johnson Amala Justin, Aswin Asokan, T. Maria Jusler Kalsingh, Manu Thomas Mathai, Neena Valecha, Jacqui Montgomery, Matthew B. Thomas, Alex Eapen

**Affiliations:** 1 ICMR-National Institute of Malaria Research, IDVC Field Unit, NIE Campus, 2nd Main Road, TNHB, Ayapakkam, Chennai, 600 077 India; 2 Department of Zoology, Madras Christian College, Tambaram, Chennai, 600 059 India; 3 ICMR-National Institute of Malaria Research, Sector 8, Dwarka, New Delhi, 110 077 India; 4 Department of Entomology, The Pennsylvania State University, University Park, PA 16802 USA

**Keywords:** Extrinsic incubation period, Microclimate, Relative humidity, Daily temperature range, *Anopheles stephensi*, Man hour density, Urban malaria

## Abstract

**Background:**

Environmental factors such as temperature, relative humidity and their daily variation influence a range of mosquito life history traits and hence, malaria transmission. The standard way of characterizing environmental factors with meteorological station data need not be the actual microclimates experienced by mosquitoes within local transmission settings.

**Methods:**

A year-long study was conducted in Chennai, India to characterize local temperature and relative humidity (RH). Data loggers (Hobos) were placed in a range of probable indoor and outdoor resting sites of *Anopheles stephensi*. Recordings were taken hourly to estimate mean temperature and RH, together with daily temperature range (DTR) and daily relative humidity range. The temperature data were used to explore the predicted variation in extrinsic incubation period (EIP) of *Plasmodium falciparum* and *Plasmodium vivax* between microhabitats and across the year.

**Results:**

Mean daily temperatures within the indoor settings were significantly warmer than those recorded outdoors. DTR in indoor environments was observed to be modest and ranged from 2 to 6 °C. Differences in EIP between microhabitats were most notable during the hottest summer months of April–June, with parasite development predicted to be impaired for tiled houses and overhead tanks. Overall, the prevailing warm and stable conditions suggest rapid parasite development rate regardless of where mosquitoes might rest. Taking account of seasonal and local environmental variation, the predicted EIP of *P. falciparum* varied from a minimum of 9.1 days to a maximum of 15.3 days, while the EIP of *P. vivax* varied from 8.0 to 24.3 days.

**Conclusions:**

This study provides a detailed picture of the actual microclimates experienced by mosquitoes in an urban slum malaria setting. The data indicate differences between microhabitats that could impact mosquito and parasite life history traits. The predicted effects for EIP are often relatively subtle, but variation between minimum and maximum EIPs can play a role in disease transmission, depending on the time of year and where mosquitoes rest. Appropriate characterization of the local microclimate conditions would be the key to fully understand the effects of environment on local transmission ecology.

**Electronic supplementary material:**

The online version of this article (10.1186/s12936-018-2342-1) contains supplementary material, which is available to authorized users.

## Background

Climate change is expected to significantly affect the global spread, intensity and distribution of malaria. It greatly influences the El Niño cycle that is known to be associated with increased risks of some diseases transmitted by mosquitoes, such as malaria, dengue and Rift Valley fever [1]. The global temperature has risen significantly over the past 100 years, with an accelerated warming trend since the mid-1950s. Elementary modeling suggests that this increase will enhance the transmission rates of mosquito-borne disease and widen its geographical distribution, with an increase in malaria, in particular, being identified as a potential impact of climate change [1]. In extratropics (Eurasia, Northern America, most of Northern Africa and Australia) malaria transmission is highly seasonal owing to temperate climatic conditions [2]. Climatic variables like temperature and relative humidity (RH) have profound effects on the life history traits of mosquitoes [3–5]. While RH affects the lifespan of mosquitoes, temperature influences the transmission dynamics of malaria by affecting the parasite development in mosquito [6, 7]. Thus, studies which consider only the effect of temperature on malaria dynamics ignoring the other key climate factors [6], such as humidity and rainfall, are likely to produce inaccurate estimates as the key climate variables are dependent on each other [8].

Majority of studies considering the effect of temperature on mosquito bionomics and malaria riskuse temperatures recorded from standard outdoor weather stations. However, as proved, they do not necessarily represent the precise temperatures experienced by vectors in local transmission settings in the field [9]. It is reported that the mosquitoes never get exposed to a mean microclimate, but to specific climatic variables in those micro-environments where they rest [10]. A variety of indoor, as well as outdoor habitats, are reported to act as mosquito resting sites. In addition, the temperature can vary greatly between indoor and outdoor environments and also strongly influenced by local features such as house design and materials besides, vegetation cover [9]. It has been demonstrated that a small rise of 0.5° in the ambient air temperature will result in 30–100% increase in mosquito abundance [11]. The relationship between mosquito biology and temperature has been helpful in predicting temporal and spatial patterns of malaria risk [11]. Knowledge about the indoor and outdoor micro temperature spectrum will also allow predictions on the length of local gonotrophic cycles and subsequent differences in transmission intensity provided the resting nature of the local vectors is known [12].

*Anopheles stephensi*, the local urban malaria vector, rest both indoors [13–15] and outdoors [16]. It is reported that minor differences in microclimatic variables can result in marked variation in mosquito life history trait assessments and estimates of malaria transmission [10] as changes in indoor microclimate reportedly affects the parasite development rates [17]. The significance of resting microclimate and daily temperature range (DTR) on malaria transmission is well documented in a study on climate and malaria transmission [18].

The different roof pattern/structure types of houses in Chennai are asbestos, thatched, concrete and tiled. A pilot study carried out in these structure types for a brief period of 3 months indicated indoor temperatures were warmer and stable than those recorded from outdoor environments [9]. Furthermore, thatched structures were observed to record less temperature compared to other structure types. However, the relative humidity profile and its monthly variations over a year both indoors and outdoors were not known which otherwise play a significant role in the resting preference and survival of the vector mosquitoes.

The biological processes are expected to be faster under fluctuating low temperatures but slower under fluctuating high temperatures, thus highlights the effect of diurnal temperature variation in various biological processes as established in many life attributes of organisms like insects including vector–pathogen relationships [10]. Mordecai et al. had developed a more realistic model with ecological assumptions about the thermal physiology of insects [19]. Also, Murdock et al. tested empirically the effects of small shifts in thermal profile on parasite prevalence, parasite intensity and mosquito mortality, substantially decreasing the overall vectorial capacity [20]. However, there was no measure of real-world conditions of the microclimate profile of what actually the vector experiences in the precise local transmission setting to prove those experimental works. Hence, for the first time, the current year-long study was designed to understand the microclimate profile (temperature and relative humidity) of various structure types in indoor and outdoor environment where mosquitoes are presumed to be resting in an endemic, malarious urban setting to derive accurate estimates of extrinsic incubation periods (EIP) of *Plasmodium vivax* and *Plasmodium falciparum.*

## Methods

### Field site and sampling rationale

**Fig. 1:**
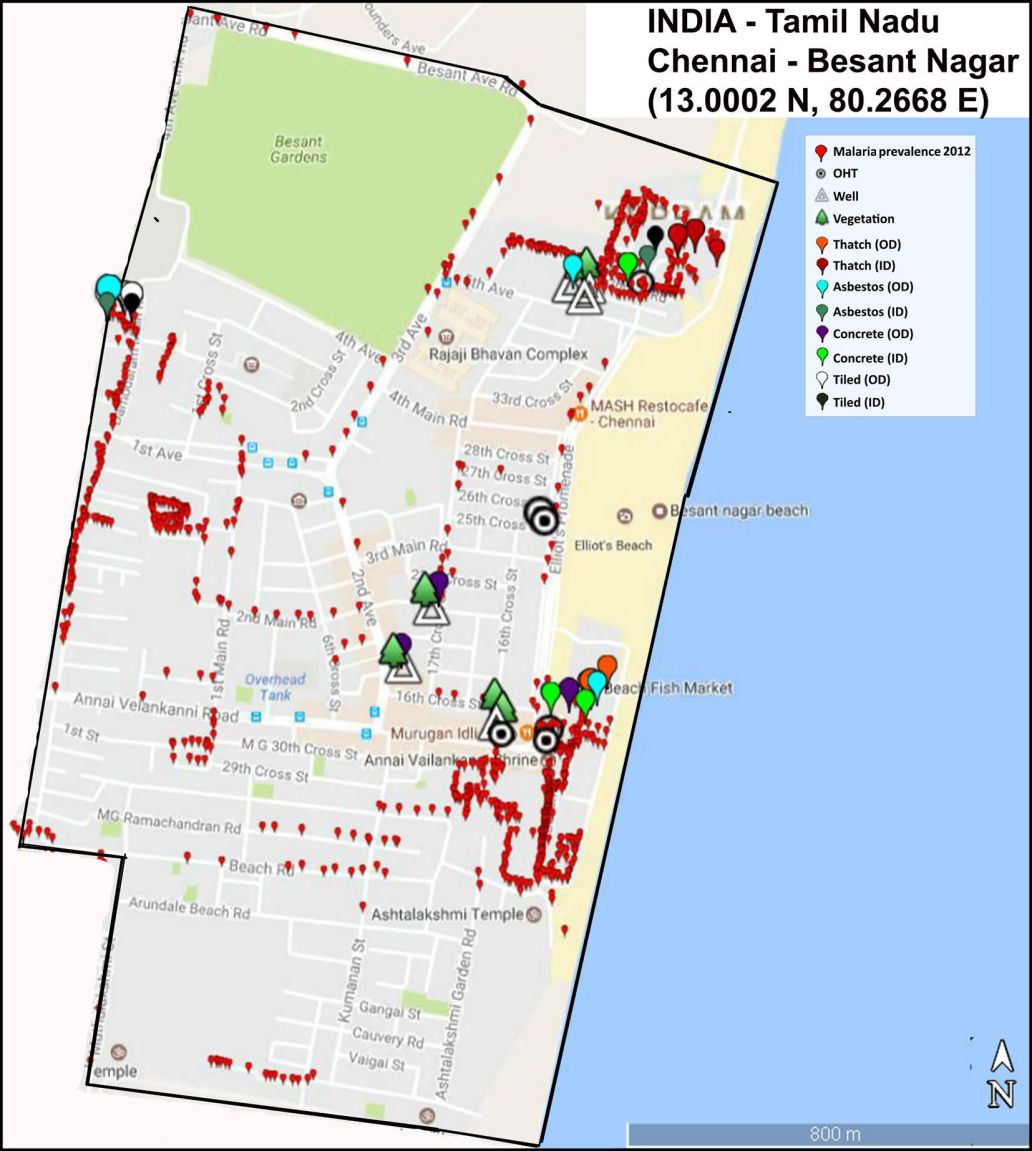
Study area indicating temperature/RH data logger (HOBO) placement locations, and malaria prevalence during the study period

The catchment area of Besant Nagar clinic (13.0002°N, 80.2668°E) was selected for the study based on the malaria prevalence during the period, 2006–2012 obtained from the Regional Office for Health and Family Welfare (ROH and FW), Besant Nagar, Chennai. Appropriate sites were selected for year-long environmental monitoring to cover all the possible microclimatic regimes of the various structure types in indoor and outdoor environments of the study area (Fig. 1). The map of the study area with hobo locations was generated with the help of Google Earth Pro v7.1. These included the common household roof structure types like thatched, tiled, asbestos and concrete material besides, other outdoor structures like overhead tanks (OHTs) and wells, the potential breeding habitats of *An. stephensi* and the vegetation. The study was conducted from November 2012 to October 2013 in Besant Nagar, a residential area with slums adjacent to the seashore in the south-eastern part of Chennai; distinctly characterized by its meso-endemic perennial transmission of malaria, predominantly *P. vivax.*

### Monitoring of microclimate (temperature and relative humidity) variables

Microclimatic temperature and relative humidity (RH) of the resting environments that adult *An. stephensi* are presumed to experience were recorded using 42 temperature/RH data loggers (Onset HOBO U10-003) on an hourly basis. They were equally distributed among seven structure types with six replicates including indoor and outdoor environments of human dwellings with varied roof structures and three selected outdoor resting sites of OHTs, wells and vegetation. Back-up for each structure type was also included in case of any missing or malfunction readings. Prior to the placement of data loggers, informed consent was obtained from the household members for keeping them year-long at the same location without any disturbance. Hobos were labelled with numbers (1–42) and distributed in the field site with details of each location documented for quick reference/identification. The coordinates of each logger were recorded using a Global Positioning System (GPS: Garmin-version 2.40). Hobos were either hung on the nails available on the walls or were kept over an open, horizontal flat surface, one to two feet down, from the roof for indoor and similarly in outdoor structures. Care was taken to place the data loggers at locations away from sources of heat and moisture in houses, such as laundry, showers and cooking area, which would provide inaccurate readings of a particular area [21]. In wells, the HOBOs were hung above the normal and expected increase of water level during monsoon period after confirming it with the house owners. Similarly, in the OHTs, the HOBOs were placed above the water level to avoid submerging of the HOBOs. Routine monthly visits were made during the study period in the forenoons and the data from each logger was downloaded on to a laptop with a software, Hoboware Lite (Ver. 3.2.1). While downloading, the data was checked for any errors; an abnormal or large number of missing readings and also the functioning besides, the battery conditions of the HOBO data loggers. If anything was found abnormal, the respective data was excluded and the HOBO was reset, ran for a short time and checked for readings. If the readings were recorded properly, the device was re-launched and placed at the same location. Malfunctioning or missing loggers were replaced with new backups. Further, after obtaining a year-long dataset, data cleaning was performed to remove any missing, abnormal values. The excluded data from the malfunctioned hobos were compensated by replacing them with the corresponding data points from the backup hobos placed in the respective structure types.

### Malaria prevalence, man-hour density (MHD) and rainfall of the study site

The monthly malaria prevalence for the study period at the study site was obtained from Regional Office for Health and Family Welfare, Government of India at Besant Nagar, Chennai. The man-hour density (MHD) for the study period was obtained from a parallel study of the fortnightly collections of the adult female *An. stephensi* mosquitoes in cattle sheds during the dusk period. The monthly MHD of *An. stephensi* was calculated by dividing the total number of female mosquitoes collected by total time spent for a particular month for one hour period i.e., (Total female *An. stephensi* collected/Total time spent) × 60 min [22]. The monthly rainfall data was obtained from Regional Meteorological Centre at Nungambakkam, Chennai, India.

### Data analysis

The downloaded data points were arranged on the basis of individual hobos, day and month wise and also structure types. The maximum, minimum and mean values of the temperature and RH of structure types were calculated based on hour, day and logger wise besides, on monthly basis. Daily temperature range (DTR) is considered as the difference between the highest and lowest values of temperatures, recorded during a day while daily relative humidity range (DRHR) is the difference between highest and lowest values of relative humidity recorded during a day. The monthly mean DTR was calculated as the mean of all DTR values of the corresponding month. The readings logged between 6.00 and 17.59 h were considered as day-time/diurnal readings when anophelines, in general, are considered to be primarily resting and those recorded during 18.00 and 5.59 h were night-time/nocturnal readings when they are in an active phase [23]. Hour, month, indoor–outdoor, structure type and season-wise differences in temperature, RH, DTR and DRHR as well as their diurnal and nocturnal variations were analysed statistically (Independent t-test and ANOVA). The environmental monitoring data was processed by IBM SPSS version 21. Further, extrinsic incubation period (EIP) was derived from the longitudinal temperature readings observed in the study site. EIP is defined as the interval between the acquisition of parasite by the vector and the vector’s ability to transmit the parasite to other susceptible vertebrate hosts. It is calculated as the reciprocal of parasite development rate (PDR). PDR of *P. vivax and P. falciparum* was estimated using the following equation [9].

$${\text{PDR}}_{{{\text{falciparum }}({\text{T}})}} \, = \,0.000 1 1 2 {\text{T }}({\text{T}}\, - \, 1 5. 3 8 4) \, \sqrt {( 3 5\, - \,{\text{T}})} ,$$

$${\text{PDR}}_{{{\text{vivax }}({\text{T}})}} \, = \,0.000 1 2 6 {\text{T }}({\text{T}}\, - \, 1 4. 2 4 4) \, \sqrt {( 3 4. 4\, - \,{\text{T}})} \quad \, ({\text{R}}^{ 2} \, = \,0. 8 9 7).$$

According to the thermodynamic model, 34.4 °C (*P. vivax)* and 35 °C *(P. falciparum)* were considered as the critical maximum temperature wherein parasite developments were assumed to be blocked if mean temperatures exceeded the respective CT_max_ [9]. Since the temperature data points above those maximum limits (43 data points out of 4015 in the present dataset) were assumed to generate invalid estimates of EIP, they were excluded from the EIP estimation analysis to make the data more reliable and accurate. Further, the relationships among mean temperature, RH, DTR, DRHR, rainfall, MHD, EIP and monthly malaria prevalence were analysed using Pearson correlation test.

## Results

### Diversity of microclimatic (temperature and RH) profile among structure types of indoor and outdoor environments

The microclimate (temperature and RH) profile observed during the year-long study revealed variations among the structure types in indoor and outdoor environments. The recorded ambient temperature and RH represent the actual microclimate conditions experienced in these structure types with a wide range of DTR, DRHR and also variations in diurnal and nocturnal temperature in all the structure types found indoors and outdoors.

### Variations in temperature and RH observed based on hours, months and seasons

**Fig. 2:**
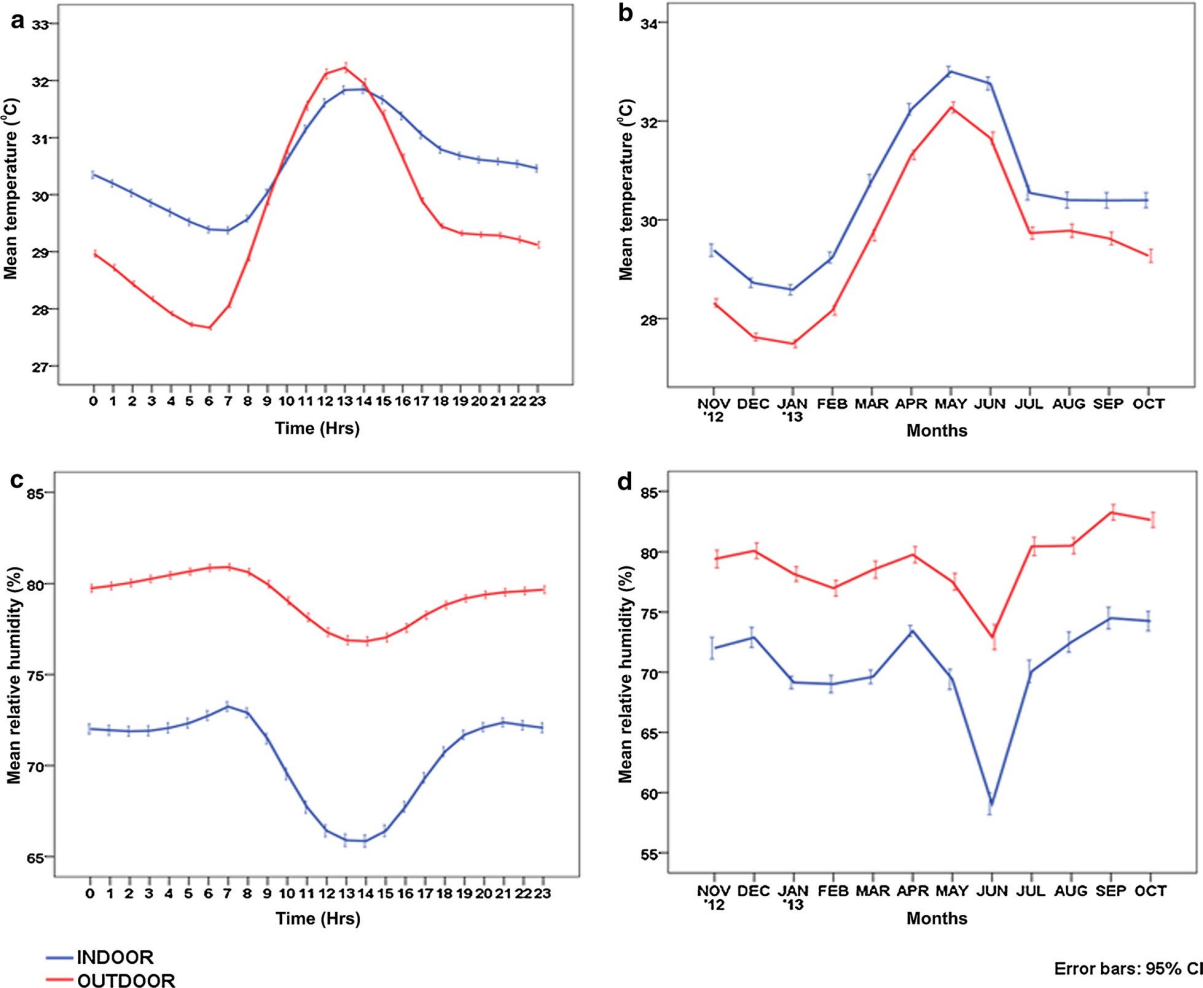
Hour and month wise variations in mean temperature (**a**, **b**) and relative humidity (**c**, **d**) observed in the study site

The average hourly, monthly temperature profile and RH recorded by the data loggers indicated that indoor temperatures were warmer when compared to outdoors with less humidity (Fig. 2). Among the structure types of roof materials (households), the indoor mean temperature ranged from 27.63 ± 0.62 °C in thatched (Jan. ‘13) to 33.66 ± 1.05 °C in tiled structure (May ‘13). In the outdoor structures of households, the corresponding mean temperature ranged from 26.64 ± 0.44 °C in thatched (Jan. ‘13) to 33.22 ± 0.93 °C in tiled (May ‘13). Thatched (indoor and outdoor) structure was cooler with a low-temperature profile in contrast to tiled structure, with a warmer ambient environment of high temperature. In the case of other outdoor structures (OHTs, wells and vegetation), the lowest temperature of 26.72 ± 0.8 °C (Jan. ‘13) was observed in vegetation and the highest of 34.15 ± 2.29 °C (May ‘13) in the overhead tank (Table 1). The DTR observed a wide temperature range in other outdoor structures ranging from 0.89 °C (Dec. ‘12) in wells to 14.62 °C (Mar. ‘13) in OHTs (Table 1). In indoors, DTR ranged from 1.93 °C (Sept. ‘13) in concrete to 7.07 °C (June ‘13) in thatched structure whereas, in outdoors, it ranged from 2.3 °C (Dec. ‘12) in concrete to 12.01 °C (Feb. ‘13) in asbestos structure.

**Table 1:** Month-wise variations in temperature and relative humidity (RH) of different structure types across indoor and outdoor environments

Structure type	Indoor/outdoor	N	Mean	Nov-12	Dec-12	Jan-13	Feb-13	Mar-13	Apr-13	May-13	Jun-13	Jul-13	Aug-13	Sep-13	Oct-13
Asbestos	Indoor	3	Temp (°C)	29.99 ± 1.12	29.31 ± 0.97	29.23 ± 1.07	29.81 ± 0.96	31.27 ± 0.87	32.55 ± 0.6	33.16 ± 0.71	32.91 ± 1.22	30.76 ± 1.39	30.37 ± 1.73	30.5 ± 1.73	30.89 ± 1.65
			DTR (°C)	5.09	4.33	4.61	4.32	4.47	4.75	4.43	4.78	3.94	4.47	4.35	4.44
			RH (%)	69.79 ± 8.87	70.44 ± 8.32	66.56 ± 4.72	67.04 ± 6.56	68.01 ± 4.81	72.3 ± 3.05	69.12 ± 7.55	59.54 ± 8.15	70.28 ± 8.51	73.63 ± 7.41	75.3 ± 8.08	73.54 ± 7.61
	Outdoor	3	Temp (°C)	28.53 ± 0.97	27.9 ± 0.9	27.88 ± 0.82	29.12 ± 1.13	30.51 ± 1.3	31.67 ± 1.05	32.48 ± 1.1	32.01 ± 1.34	30 ± 1.68	29.96 ± 1.56	30.07 ± 1.56	29.78 ± 1.75
			DTR (°C)	7.31	8.21	9.41	12.01	10.5	7.38	7.38	7	7.11	6.41	6.26	6.83
			RH (%)	73.49 ± 7.22	73.99 ± 8.49	71.36 ± 5.29	69.27 ± 7.84	70.13 ± 7.06	75.65 ± 4.9	72.21 ± 8.24	62.55 ± 8.16	73.77 ± 8.43	74.6 ± 6.96	76.59 ± 7.54	77.36 ± 7.33
Concrete	Indoor	3	Temp (°C)	29.58 ± 1.04	28.86 ± 0.64	28.82 ± 0.68	29.4 ± 0.73	30.91 ± 1.16	32.31 ± 1.24	32.97 ± 1.14	32.79 ± 1.16	31 ± 1.2	30.51 ± 1.69	30.4 ± 1.63	30.62 ± 1.34
			DTR (°C)	2.47	2.07	2.69	2.65	2.69	2.47	2.48	3.17	2.19	2.13	1.93	2.22
			RH (%)	73.1 ± 8.13	74.16 ± 7.25	69.97 ± 4.72	69.15 ± 6.45	69.96 ± 5.31	73.87 ± 4.27	69.6 ± 8.54	57.66 ± 8.01	67.62 ± 7.97	71.73 ± 8.77	73.62 ± 8.67	72.99 ± 7.41
	Outdoor	3	Temp (°C)	27.88 ± 0.93	27.17 ± 0.61	26.84 ± 0.71	27.42 ± 0.73	28.9 ± 0.98	30.7 ± 0.72	31.78 ± 0.85	31.68 ± 1.27	29.47 ± 1.26	29.52 ± 1.3	29.37 ± 1.29	28.89 ± 1.38
			DTR (°C)	3.52	2.3	2.79	3.16	3.44	2.84	3.25	4.3	3.32	3.13	2.97	2.76
			RH (%)	74.1 ± 8.12	75.92 ± 8.02	73.2 ± 5.77	73.6 ± 6.63	75.29 ± 5.48	78.76 ± 4.34	73.82 ± 8.5	62.07 ± 8.05	73.37 ± 7.37	74.36 ± 6.98	78.11 ± 7.33	79.21 ± 6.84
Thatched	Indoor	3	Temp (°C)	28.53 ± 0.95	27.9 ± 0.6	27.63 ± 0.62	28.2 ± 0.62	29.69 ± 0.77	31.22 ± 0.64	32.21 ± 0.72	32.16 ± 1.22	29.96 ± 1.27	30.15 ± 1.44	30.08 ± 1.33	29.83 ± 1.41
			DTR (°C)	5.01	4.1	4.51	5.01	5.35	4.86	5.5	7.07	4.77	5.32	5.01	4.27
			RH (%)	72.33 ± 8.3	74.06 ± 7.27	70.81 ± 4.16	71.77 ± 6.16	72.83 ± 4.6	76.77 ± 2.69	72.07 ± 7.71	60.62 ± 8.94	71.7 ± 9.36	72.95 ± 7.67	74.82 ± 8.05	75.5 ± 7.24
	Outdoor	3	Temp (°C)	28.16 ± 1.04	26.94 ± 0.46	26.64 ± 0.44	27.19 ± 0.5	29.29 ± 1.17	31.46 ± 0.66	32.27 ± 0.87	31.78 ± 1.37	29.68 ± 1.71	29.62 ± 1.53	29.61 ± 1.51	29.04 ± 1.59
			DTR (°C)	5.54	3.47	4.39	4.74	6.33	6.9	6.65	7.59	6.78	6.45	5.94	5.11
			RH (%)	74.01 ± 7.5	77.48 ± 6.16	78.06 ± 4.87	78 ± 5.19	76.87 ± 4.09	77.06 ± 2.68	74.56 ± 6.8	64.46 ± 9.21	75.77 ± 11.98	77.15 ± 8.5	80.24 ± 9.47	81.45 ± 8.6
Tiled	Indoor	3	Temp (°C)	29.44 ± 1.25	28.82 ± 0.9	28.66 ± 0.94	29.54 ± 1.13	31.31 ± 1.27	32.86 ± 1.17	33.66 ± 1.05	33.16 ± 1.34	30.45 ± 1.56	30.56 ± 1.47	30.58 ± 1.38	30.24 ± 1.42
			DTR (°C)	3.28	3.12	3.21	3.4	3.95	3.36	3.5	4.14	3.07	3.38	3.27	2.75
			RH (%)	72.79 ± 9.21	72.88 ± 9.14	69.25 ± 5.87	68.1 ± 7	67.67 ± 6.21	70.77 ± 5	66.84 ± 8.62	58.45 ± 9.82	70.67 ± 10.31	71.69 ± 8.98	74.25 ± 10.1	74.96 ± 9.49
	Outdoor	3	Temp (°C)	28.43 ± 0.97	27.83 ± 0.57	27.65 ± 0.59	28.91 ± 0.76	30.62 ± 1.05	32.32 ± 0.95	33.22 ± 0.93	32.91 ± 1.37	30.3 ± 1.64	30.42 ± 1.76	30.34 ± 1.56	30.04 ± 1.6
			DTR (°C)	6	5.69	6.56	7.12	6.97	5.81	7.21	9.06	7.22	7.29	6.44	6.66
			RH (%)	76.94 ± 8.54	78.45 ± 8.22	75.53 ± 6.47	72.62 ± 6.8	72.82 ± 4.95	75.15 ± 2.33	71.32 ± 7.33	62.36 ± 8.89	74.09 ± 9.36	75.79 ± 8.25	78.93 ± 9.11	80.36 ± 8.39
OHT	Other outdoor	6	Temp (°C)	29.75 ± 1.15	29.14 ± 1.3	29.28 ± 0.78	30 ± 1.08	31.74 ± 1.68	32.99 ± 1.7	34.15 ± 2.29	33.62 ± 2.14	31.42 ± 2.37	31.88 ± 2.6	31.4 ± 2.47	31.18 ± 2.48
			DTR (°C)	10.4	11.19	12.07	14.21	14.62	12.4	13.81	13.21	11.73	14.56	12.82	13.56
			RH (%)	80.49 ± 9.89	80.41 ± 9.09	75.67 ± 6.67	76.59 ± 9.92	81.9 ± 10.07	84.08 ± 10.15	79.49 ± 13.03	85.41 ± 13.57	75.03 ± 5.76	76.16 ± 9.87	81.5 ± 10.39	78.17 ± 9
Well	Other outdoor	6	Temp (°C)	27.44 ± 0.77	27.1 ± 0.51	26.96 ± 0.55	27.38 ± 0.71	28.36 ± 0.84	30.01 ± 0.89	30.74 ± 0.91	29.96 ± 1.12	28.66 ± 0.97	28.42 ± 0.91	28.17 ± 0.93	27.99 ± 0.92
			DTR (°C)	1.08	0.89	0.93	1.19	1.41	2.82	3.88	3.35	2.14	2.15	1.07	0.96
			RH (%)	94.26 ± 7.43	90.86 ± 8.3	91.14 ± 8.77	88.01 ± 6.52	84.97 ± 16.67	83 ± 17.97	88.61 ± 7.67	91 ± 6.68	95.09 ± 4.97	93.98 ± 4.47	94.83 ± 4.12	96.14 ± 3.44
Vegetation	Other outdoor	6	Temp (°C)	27.77 ± 1.12	27.01 ± 0.95	26.72 ± 0.8	27.33 ± 0.78	28.75 ± 1	30.72 ± 0.65	31.7 ± 0.84	31.32 ± 1.3	28.97 ± 1.46	28.82 ± 1.46	28.84 ± 1.44	28.4 ± 1.48
			DTR (°C)	6.65	5.27	6.36	6.49	7.24	7.58	7.29	8.41	6.26	6.02	6.11	5.27
			RH (%)	75.34 ± 8.72	77.4 ± 7.78	74.88 ± 6.69	74.87 ± 7.69	78.43 ± 7.7	78.98 ± 4.52	73.61 ± 7.95	67.77 ± 11	84.89 ± 10.21	81.5 ± 7.33	83.03 ± 5.25	81.07 ± 4.94

However, RH observed a different picture in indoor household structure types ranging from 57.66 ± 8.01% (June ‘13) in the concrete structure to 76.77 ± 2.69% (Apr. ‘13) in thatched structures. Similarly, RH in outdoor household structure types ranged from 62.07 ± 8.05% (June ‘13) in concrete to 81.45 ± 8.6% (Oct. ‘13) in thatched structures. Among all structure types, thatched structures were more humid, both indoors and outdoors whereas, concrete structures experienced the lowest humidity. In other outdoor structures, RH was lowest in vegetation (67.77 ± 11%) during June ‘13 and highest in well (96.14 ± 3.44%) during Oct. ’13 (Table 1). The mean temperature was warmer and obvious during the early part of the day (morning) and later remained almost constant (evening and night) in indoors (Fig. 2a, b). However, outdoors were more humid than indoors and varied drastically during the 24 h period (Fig. 2c) as well as throughout the study period (Fig. 2d).

**Fig. 3:**
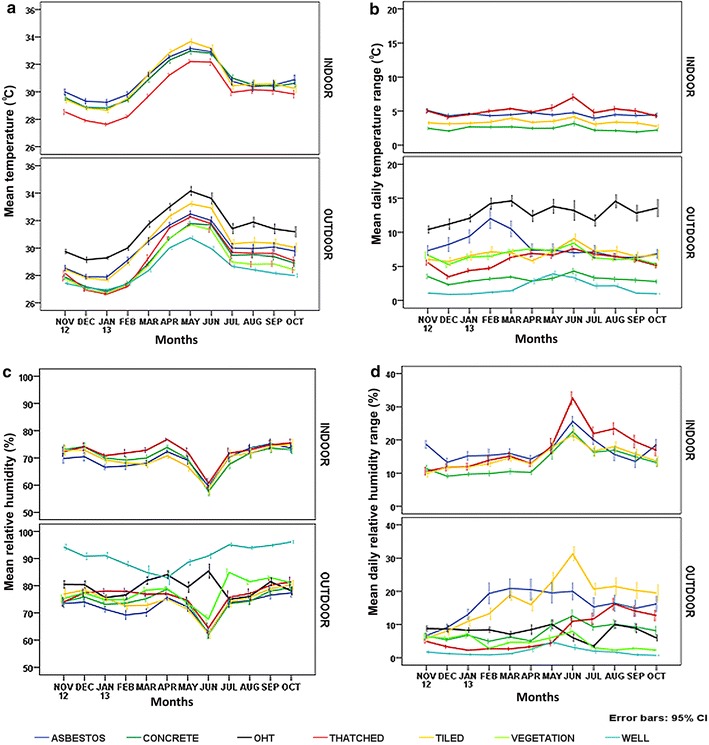
Structure type wise variations in mean temperature (**a**), mean daily temperature range (**b**) mean relative humidity (**c**) and mean daily relative humidity range (**d**) in indoor and outdoor environments

Similarly, month wise observation of microclimate profile (temperature and humidity) experienced by indoor and outdoor environments among various structure types are detailed in Table 1 and Fig. 3. In indoors, thatched structure observed the minimum temperature profile throughout the study period whereas, tiled exhibited the highest temperature in May ‘13 with fluctuations during the initial (Nov. ‘12 to Mar. ‘13) and later part (June ‘13 to Oct. ‘13). In outdoors, the lowest temperature was recorded for thatched (Jan. ‘13) and the maximum temperature was consistent for OHTs (Fig. 3a). Mean DTR had maximum variations by and large for thatched and the least for concrete in indoor structures. Similarly, the mean DTR had the highest variations in OHTs and the lowest in wells among all other outdoor structures (Fig. 3b). The mean RH recorded, indicated a uniform pattern in indoors for all structure types, however in outdoors, wells recorded the maximum (Fig. 3c). The mean DRHR indicated maximum for indoor thatched structure whereas, in outdoors, tiled structure recorded the maximum (Fig. 3d).

When the mean values of each of the variables (temperature, RH, DTR, DRHR) were compared across different combinations like (i) all indoors versus all outdoors (ii) indoors of human dwellings/households versus the corresponding outdoor environments and (iii) human dwellings in general versus outdoor resting habitats alone, it was observed that in all the three combinations, mean temperature of indoors and human dwellings in general (indoor and outdoor) were observed to be significantly higher compared to outdoors (Table 2). However, RH and DTR of all household structure types (indoor and outdoor) were low whereas, all outdoor structure types were significantly higher among the all-different combinations (p < 0.001).

**Table 2:** Variations in temperature and relative humidity (RH) observed in all structure types of indoor and outdoor environments

Type	No.	Temperature (°C)		RH (%)		DTR (°C)		DRHR (%)	
		Mean (min, max)	95% CI	Mean (min, max)	95% CI	Mean (min, max)	95% CI	Mean (min, max)	95% CI
All indoors	4	30.54 (25.56, 35.92)	30.48–30.59	70.51 (40.52, 94.37)	70.25–70.76	3.84 (0.4, 11.55)	3.78–3.89	15.64 (0.77, 54.61)	15.39–15.88
All outdoors	7	29.58 (24.45, 42.36)	29.54–29.62	79.22 (39.38, 105.24)	79.01–79.44	6.57 (0.1, 35.15)	6.47–6.67	7.84 (0, 57.2)	7.67–8.02
Indoors (human dwellings only)	4	30.54 (25.56, 35.92)	30.48–30.59	70.51 (40.52, 94.37)	70.25–70.76	3.84 (0.4, 11.55)	3.78–3.89	15.64 (0.77, 54.61)	15.39–15.88
Outdoors (human dwellings only)	4	29.69 (24.58, 36.59)	29.63–29.75	74.42 (45.98, 100.09)	74.17–74.67	5.9 (0.39, 25.82)	5.85–6.06	12.23 (0.11, 57.2)	11.91–12.56
Human dwellings	4	30.11 (24.58, 36.59)	30.10–30.12	72.47 (40.52, 100.09)	72.42–72.51	4.89 (0.39, 25.82)	4.82–4.95	13.94 (0.11, 57.2)	13.73–14.15
Exclusive outdoors (OHT, well, vegetation)	3	29.51 (24.45, 42.36)	29.45–29.57	82.6 (39.38, 105.24)	82.32–82.88	6.99 (0.1, 35.15)	6.84–7.14	4.76 (0, 50.89)	4.61–4.91

Similarly, when the structure wise difference in the indoor and outdoor environment was analysed for temperature, RH, DTR and DRHR (Table 3), it was observed that all the variables were significantly different in the indoor and outdoor environment and also between indoors and outdoors of the different structure types (p < 0.001). When the mean temperatures of OHTs were compared with the temperature of all indoors in general and also with each indoor structure type (tiled, asbestos, concrete, thatched), it showed a significant difference (p < 0.001). Further, when the mean temperature of wells when compared with the temperatures of all indoors together and with each indoor structure type, it showed a significant difference (p < 0.001) in the temperature profile.

**Table 3:** Mean, minimum, maximum temperature and relative humidity (RH) variations of different structure types in indoor and outdoor environments

Structure	Indoor/outdoor	Temperature (°C)		RH (%)		DTR (°C)		DRHR (%)	
		Mean (min, max)	95% CI	Mean (min, max)	95% CI	Mean (min, max)	95% CI	Mean (min, max)	95% CI
Asbestos	Indoor	30.89 (25.86, 35.49)	30.79–31.00	69.67 (44.76, 90.66)	69.55–69.79	4.45 (0.69, 8.33)	4.42–4.57	16.95 (0.77, 43.24)	16.47–17.44
	Outdoor	29.98 (25.36, 36.59)	29.94–30.03	72.61 (45.98, 93.75)	72.48–72.73	7.96 (0.59, 25.82)	7.68–8.24	15.97 (0.63, 57.2)	15.21–16.72
Concrete	Indoor	30.68 (26.17, 35.92)	30.58–30.79	70.3 (45.02, 92.64)	70.19–70.42	2.42 (0.4, 6.65)	2.35–2.49	13.4 (1.76, 44.41)	12.99–13.82
	Outdoor	29.13 (24.84, 34.56)	29.11–29.16	74.34 (48.54, 94.82)	74.24–74.45	3.14 (0.39, 9.77)	3.05–3.23	7.84 (0.4, 40.52)	7.49–8.20
Thatched	Indoor	29.8 (25.56, 34.3)	29.69–29.90	72.2 (45.51, 92.12)	72.08–72.32	5.06 (0.49, 11.55)	4.95–5.16	17.41 (1.79, 54.61)	16.82–17.99
	Outdoor	29.37 (24.58, 34.54)	29.33–29.40	76.27 (46.85, 100.09)	76.15–76.38	5.88 (0.59, 15.75)	5.74–6.02	7.56 (0.56, 39.27)	7.10–8.02
Tiled	Indoor	30.77 (26.46, 35.64)	30.65–30.89	69.9 (40.52, 94.37)	69.77–70.03	3.36 (0.59, 7.75)	3.27–3.45	14.78 (1.46, 44.59)	14.35–15.20
	Outdoor	30. 24 (25.57, 35.54)	30.20–30.28	74.57 (49.18, 98.31)	74.44–74.71	6.83 (0.98, 19.17)	6.63–7.03	17.44 (0.11, 54.58)	16.74–18.15
OHT	Outdoor	31.19 (24.93, 42.36)	31.15–31.24	79.13 (43.15, 103.8)	79.05–79.22	12.87 (0.98, 35.15)	12.59–13.14	7.27 (0, 44.88)	7.64–8.29
Vegetation	Outdoor	28.86 (24.45, 34.82)	28.83–28.88	77.68 (51.24, 103.73)	77.61–77.76	6.57 (0.89, 22.72)	6.44–6.69	4.65 (0.01, 50.89)	4.43–4.86
Well	Outdoor	28.48 (25.29, 32.85)	28.46–28.49	90. 93 (39.38, 105.24)	90.84–91.02	1.86 (0.1, 17.73)	1.76–1.95	1.84 (0, 46.63)	1.68–1.99

### Season-wise fluctuations in temperature, RH during day and night in various structure types of indoor and outdoor environments

It was observed that, across the months, diurnal and nocturnal temperature besides, RH had a significant difference (p < 0.001). The day temperatures were significantly higher, except concrete indoors, compared to nights (p < 0.001) across various structure types in both indoor and outdoor environments (see Additional file 1), while RH was almost the same without much difference (see Additional file 2). Further during the day-time, indoors and outdoors exhibited similar temperature pattern, whereas, during the night, the outdoor temperature was observed to be less unlike indoors, where it remained almost the same. The indoor/outdoor and structure type wise variations in the examined variables were found to be significantly different across seasons (p < 0.001). Outdoors remained more humid during day and night (see Additional file 2). When the diurnal and nocturnal variations in temperature and RH was compared season wise, it was found that except for temperature during monsoon period (Day-time = 30.29 °C; 95% CI 28.74–31.85 °C; nocturnal = 28.48 °C; 95% CI 26.84–30.12 °C), there was no significant difference in any of the variables.

It was observed that, during all seasons, the diurnal temperature was significantly different from nocturnal temperature, i.e., winter (mean diurnal temperature = 28.70 °C, 95% CI 28.67–28.72 °C; mean nocturnal temperature = 27.47 °C, 95% CI 27.45–27.49 °C); summer (mean diurnal temperature = 31.53 °C, 95% CI 31.50–31.56 °C; mean nocturnal temperature = 31.20 °C, 95% CI 31.17–31.22 °C); pre monsoon (mean diurnal temperature = 31.54 °C, 95% CI 31.50–31.57 °C; mean nocturnal temperature = 29.69 °C, 95% CI 29.67–29.71 °C); monsoon (mean diurnal temperature = 30.30 °C, 95% CI 30.27–30.33 °C; mean nocturnal temperature = 28.49 °C, 95% CI 28.47–28.51 °C). Similarly, during all seasons, the diurnal RH was significantly different from nocturnal RH, i.e., winter [mean diurnal RH = 75.80% (95% CI 75.60–75.89%); mean nocturnal RH = 76.33% (95% CI 76.24–76.42%)]; summer [mean diurnal RH = 76.31% (95% CI 76.21–76.41%); mean nocturnal RH = 76.59% (95% CI 76.49–76.69%)]; pre monsoon [mean diurnal RH = 73.13% (95% CI 72.99–73.27%); mean nocturnal RH = 76.73% (95% CI 76.61–76.84%)]; monsoon [mean diurnal RH = 78.32% (95% CI 78.21–78.43%); mean nocturnal RH = 80.42% (95% CI 80.33–80.51%)].

### Structure type variations in the extrinsic incubation period (EIP) of *Plasmodium vivax* and *Plasmodium falciparum*

**Fig. 4:**
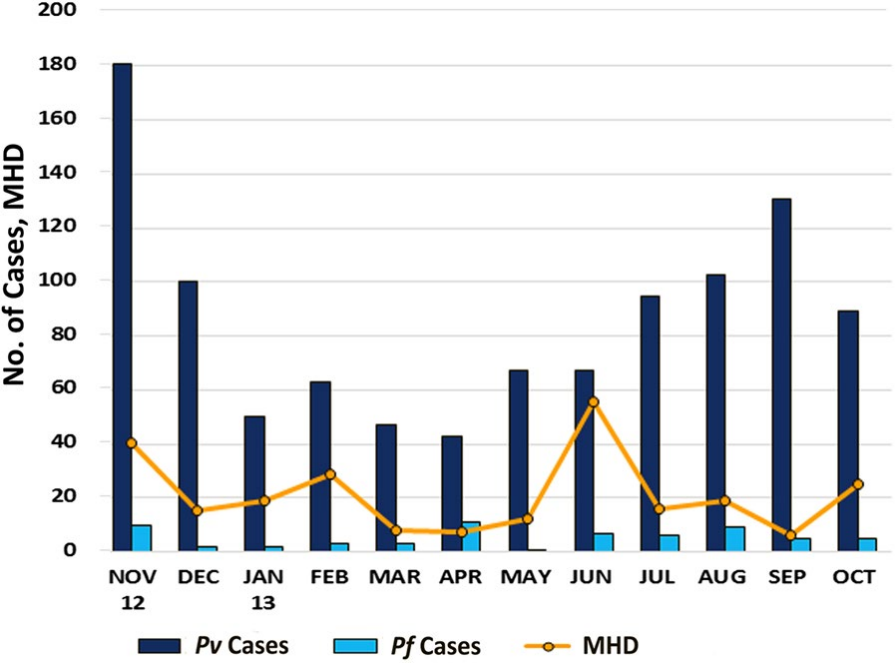
Month-wise man-hour density (MHD) of *Anopheles stephensi*, the prevalence of *Plasmodium vivax*, *Plasmodium falciparum* of the study site

**Fig. 5:**
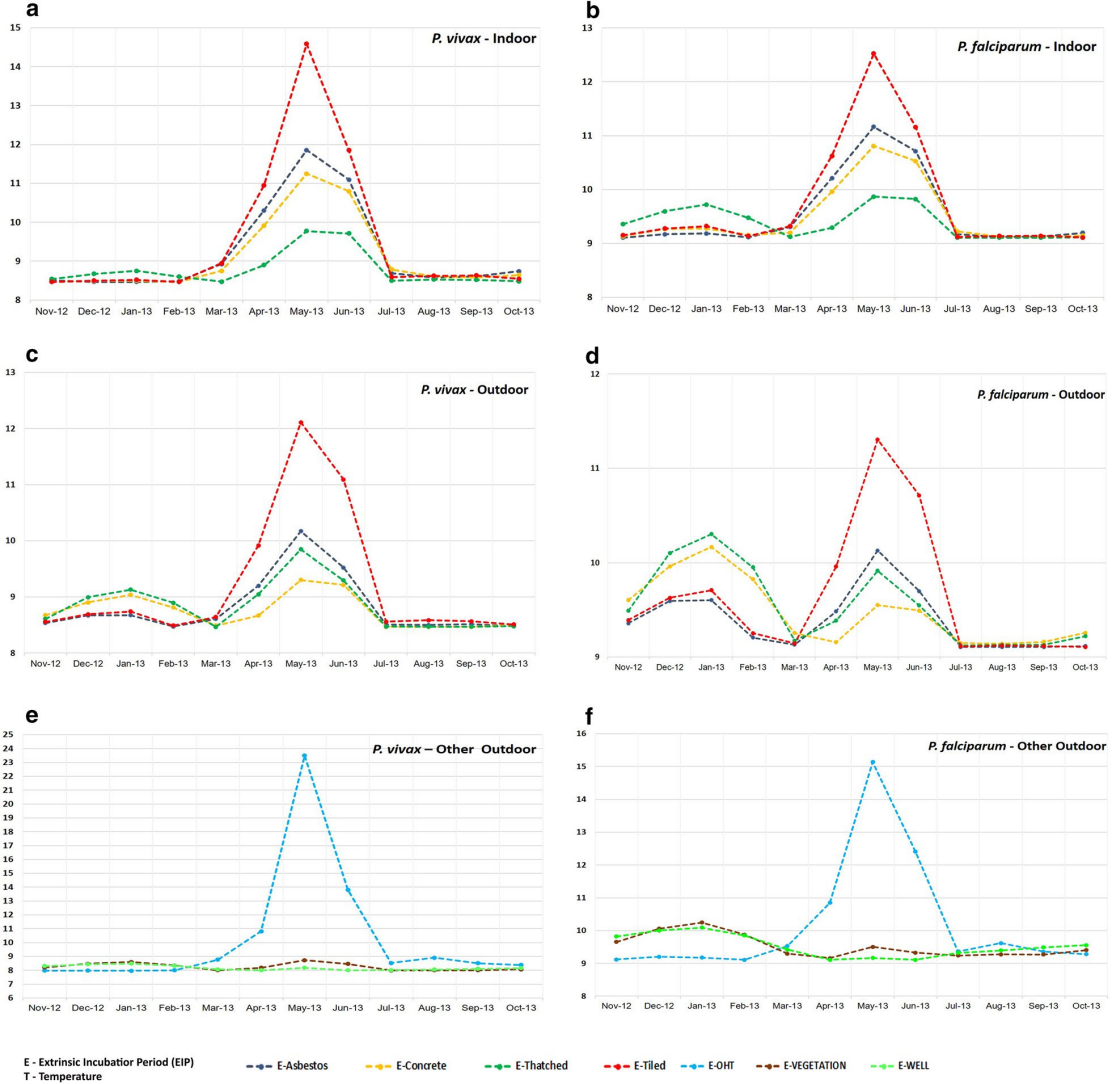
Structure type variations in extrinsic incubation periods (EIPs) of *Plasmodium vivax* (**a**, **c**) and *Plasmodium falciparum* (**b**, **d**) in indoor and outdoor environments and other outdoor structure types (overhead tanks, wells and vegetation) (**e**, **f**)

The peak prevalence of *P. vivax* and *P. falciparum* illustrates a different picture and it is quite contradictory to the man-hour density observed in the study site (Fig. 4). The estimated EIPs of various structure types in human dwellings (Fig. 5) exhibited a wide range in EIPs across indoors (*P. vivax*—7.97–14.08; *P. falciparum*—9.11–12.52) as well as outdoors (*P. vivax*—7.97–11.61; *P. falciparum*—9.11–11.30). EIPs of indoor structure types of human dwellings (except thatched) were observed to be more when compared to outdoors. However, there was no significant difference in EIP when comparisons were made among structure types of human dwellings (indoor and outdoor) and also with structure types of human dwelling outdoors against all outdoor structures (Table 4). Among the other outdoor structures, EIP of *P. vivax* was estimated to be 7.97–24.27 and for *P. falciparum*, it was 9.11–15.26 in the overhead tank (Table 4). OHT exhibited maximum temperature variations and estimates of EIP for *P. vivax* and *P. falciparum*, while wells recorded the lowest estimates of temperatures for EIP in the case of *P. falciparum*. Further, the mean EIP values of both malaria parasites derived from all indoors were compared against the EIP values of potential breeding habitats like OHTs and wells. It was observed that the estimated EIP of *P. vivax* derived from indoors (Mean EIP = 8.667) were significantly different (p = 0.040), from the EIPs derived from OHTs (Mean EIP = 10.24). However, there was no significant difference between indoors and wells. Further, when EIP values of individual indoors were compared against wells and OHTs, it was observed that EIP of *P. vivax* derived from thatched indoors (Mean EIP = 9.39) were significantly different (p = 0.048), from the EIPs derived from OHTs (Mean EIP = 10.24).

**Table 4:** Extrinsic incubation period (EIP) of *Plasmodium vivax* and *Plasmodium falciparum* among different structure types

Structure	Indoor/outdoor	*Pv* EIP range	*Pf* EIP range
Asbestos	Indoor	7.97–11.36	9.11–11.16
	Outdoor	7.97–9.67	9.11–10.13
Concrete	Indoor	7.97–10.75	9.12–10.81
	Outdoor	7.97–8.80	9.14–10.16
Thatched	Indoor	7.97–9.27	9.11–9.87
	Outdoor	7.97–9.34	9.12–10.30
Tiled	Indoor	7.97–14.08	9.11–12.52
	Outdoor	7.99–11.61	9.11–11.30
OHT	Outdoor	7.97–24.27	9.11–15.26
Vegetation	Outdoor	7.98–8.73	9.16–10.25
Well	Outdoor	8.00–8.49	9.11–10.09

### Relationship among microclimatic variables (temperature, RH and rainfall), man-hour density of *Anopheles stephensi* and malaria prevalence during the study period

The trend of temperature, RH, rainfall, DTR, MHD and average malaria prevalence (2006–2012) during the study period has been represented in Additional file 3. It was observed that, as mean temperatures increases, DTR showed a significant positive correlation (r = 0.422, p < 0.001) in general and also during different months of a year (r = 0.694, p = 0.01). However, when the data was analysed month wise, RH showed significant negative correlation with MHD (r = − 0.661, p = 0.019) and DTR (r = − 0.661, p = 0.01). Also, DTR showed significant positive correlation with DRHR (r = 0.618, p = 0.032). Further, temperature showed significant positive correlation with DTR (r = 0.694, p = 0.012) and DRHR (r = 0.755, p = 0.005).

### Structure type wise differences in temperature, relative humidity, DTR, DRHR, EIP of *Plasmodium vivax* and *Plasmodium falciparum*

**Fig. 6:**
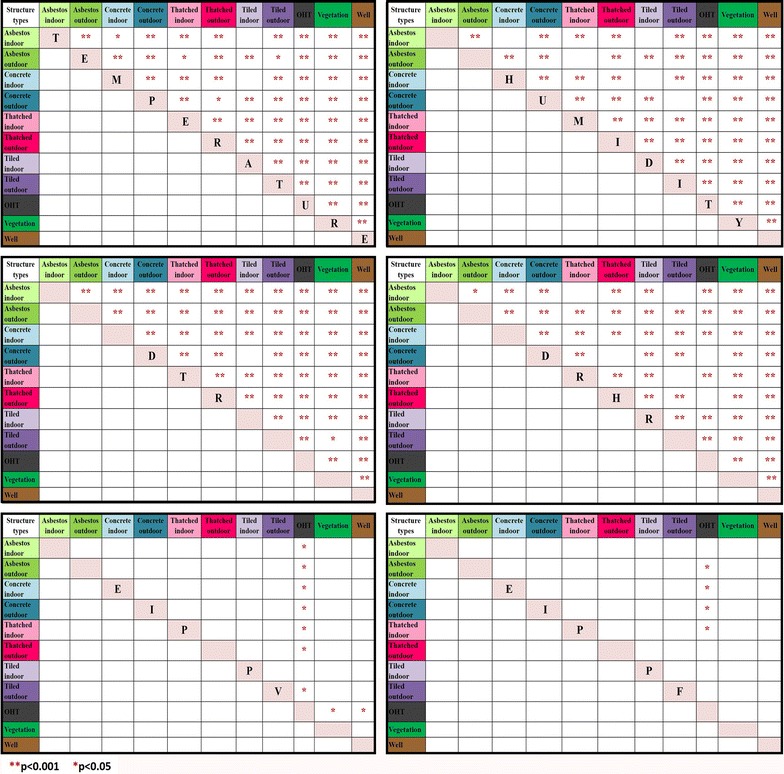
Plot indicating structure type wise significance differences in temperature, relative humidity, DTR, DRHR, EIP of *Plasmodium vivax* and *Plasmodium falciparum.* The variables are mentioned across the diagonal line. The structure types with the significant difference for respective variables are indicated with *p < 0.05 and **p < 0.001 in corresponding boxes

The various parameters of temperature, relative humidity, DTR and DRHR had a high significant difference (p < 0.001) within each structure type except few which had a less significant difference with p < 0.05 whereas some were non-significant, which are depicted in Fig. 6. Only OHT had a significant difference (p < 0.05) with other structure types when EIP of *P. vivax* and *P. falciparum* was compared with different structure types.

Mean temperature differed significantly in each structure type except indoors of asbestos, concrete and tiled. However, mean RH differed significantly in all structure types except indoors of thatched and tiled, outdoors of asbestos, concrete and tiled. Further mean DTR differed significantly in each structure type except concrete outdoor and tiled indoor. While DRHR did not differ significantly in indoors of asbestos and thatched, outdoors of concrete, thatched, tiled and OHT. EIP (*P. vivax)* did not differ significantly in different structure types except OHT which differed significantly with other structure types except tiled indoor. EIP (*P. falciparum)* also did not show any significant difference within different structure types except OHT which differed with indoors of concrete and thatched, outdoors of asbestos and concrete (Fig. 6).

## Discussion

The current study is the characterization of an urban microclimatic regime, its potential resting profiles and accordingly, the estimated EIPs of a populated, endemic malarious area with an urban slum. Since indoor,as well as outdoor microclimatic conditions, vary considerably [24], as indoor and outdoor resting of *An. stephensi* is well documented [9], a better estimate of these variations would reduce the discrepancies and errors in predicting the associations of the former with the environment or health-related variables [21]. It has been reported that differences in indoor and outdoor environments alter the limits and the intensity of malaria transmission [24, 25]. In the absence of an elaborate information on resting preferences of the local vector species [9], capturing a broad spectrum of its presumed resting profiles and accordingly the estimated EIPs provides the potential benefits to the local vector surveillance. Most of the laboratory-based experiments maintain constant temperature and humidity. The average temperature often recorded is from any outdoor weather station [26]. Hence, the present finding can be used for deriving potential intricacies of the temperature profile for laboratory-based realistic experiments to derive appropriate solutions against local malaria transmission.

Most *Plasmodium* species complete sporogonic development at constant temperatures between 16 and 30 °C and have an optimal growth rate from 21 to 28 °C [27]. This may be the reason for the higher EIPs in the present study where temperatures exceeded the optimum. Small changes in temperature had been reported to produce potentially large effects on the EIP and thereby transmission intensity [9]. Indoors exhibited higher mean temperatures and shorter DTRs compared to outdoors. Higher indoor temperature compared to outdoor was reported elsewhere [12, 18] and attributed to their general buffering nature [24]. Outdoor mean EIPs are likely to be affected by the observed readings of overhead tank, a potential breeding habitat of the local vector [28] which experienced highest temperature and DTR.

It has been reported that temperature has the potential to affect or alter the toxicity of chemicals used for ITNs, LLINs and IRS, and also the chemical release besides, mosquito response to odor-baited traps. It is also known that susceptible mosquitoes could be more resistant during cooler night-time periods [29]. Hodjati and Curtis showed that resistant *An. stephensi* mosquitoes were more susceptible to permethrin at 16 and 37 °C, compared to 22 and 28 °C where all mosquitoes survived the exposure [30]. *Anopheles stephensi*, the primary vector of urban malaria in Chennai, is actively host-seeking and blood feeding/foraging from dusk until dawn. In this context, characterizing the diurnal and nocturnal microclimate temperature profile in different resting structures would reveal the efficacy and the impact of such repellents to reduce/eliminate vector mosquitoes.

In the present study, the predicted EIP of *P. falciparum* ranged 9.1–15.3 days and EIP of *P. vivax* ranged 8.0–24.3 days. It may be noted that thatched is one of the major human dwelling structure types where the vectors prefer resting in Besant Nagar [22]. Therefore, in areas where IRS is employed for interventions invector control programme, thatched structures may be focused with effective coverage.

EIP curves or patterns of both *P. vivax* and *P. falciparum* were almost parallel in the present study as reported elsewhere [4]. Similar to the previous reports, month-wise EIP of *P. falciparum* were higher compared to *P. vivax* [31]. When temperature increased, EIPs of *P. vivax* and *P. falciparum* showed a similar trend, in human dwellings and exclusive outdoors in the study site coupled with a decline in malaria prevalence. Fluctuations around warmer temperatures are reported to decrease the parasite development, increasing the EIP and will have a negative effect on vector competence, resulting in less number of malaria cases [12, 32]. However, the prevailing warm and stable conditions (mean temperatures and DTRs rarely lesser than 27 and 5 °C respectively) in general in the study area suggest rapid parasite development rate throughout the year. This highlights the importance of active vector surveillance throughout the year.

Temperature and precipitations are important climate factors in building up mosquito populations and disease transmission dynamics. According to the Intergovernmental Panel on Climate Change, the global average temperature has increased by ~ 0.6% over the past 35 years, and the variations in precipitations have increased [33]. In Chennai, an increase of ~ 2 °C temperature was observed irrespective of the seasons over the last 10 years. Warmer temperatures and high humidity favour an increase in the longevity of adult mosquitoes and shorten the parasite development within the vector and its blood feeding intervals thus leading to increased transmission intensity. In contrast, RH has decreased by ~ 4% during peak summer months of April/May, with a further decrease of ~ 7–10% during winter seasons over last 10 years. Higher temperatures (≥ 35 °C) depending on the vector species tend to decrease disease risk because they can limit mosquito survival. Correspondingly, future climate change might further affect malaria burden and other vector-borne diseases like dengue. Therefore, understanding the ectotherm ecology of the ambient environment of mosquito vectors and deriving EIP values would provide novel ideas on how to quantify the impact change on vector mosquitoes and the disease risk. The present study indicates that EIP model based on the microtemperature of the ambient environment will also provide true predictions of the disease transmission potential in areas with low/high disease burden. Further, the finding also implies that the regular monitoring of the microclimate profile of an area will aid to identify and target the ideal habitat/area for intensified vector surveillance to keep the local transmission under check.

A few limitations of this study is considered worth mentioning. Cattle sheds were found to harbour resting anophelines in the study site. In spite of repeated persuasions, permission was denied to place hobos since the cattle shed owners were reluctant to permit and their disagreement was expressed loud and clear. These contingencies are bound to happen in a highly populated urbanized metropolitan city. The above social constraints may be due to the poor educational status of the slum dwellers and can be managed only with the help of intensive, regular awareness programmes as cattle rearing for milk is a source of living for the economically weaker groups. Another limitation is that the confounding variables such as the number and activity of the occupants in a particular structure type which may affect the temperature and RH in that space, have not been considered. In real-world conditions, it is operationally difficult to take account of those details of inhabitants on a daily, weekly or monthly basis in a metropolitan city with high population density and floating nature. Also, a few RH readings were found to be exceeding the maximum point. This might happen as the water level rises during rainy seasons or rarely while replenishing water in the overhead tanks, and can touch or submerge the HOBO for some time.

The study successfully illustrated the temperature and RH profile of the presumed adult resting sites (roof structures). The monthly, as well as structure type wise estimates of microclimatic temperatures and RH, can act as robust predictors to understand transmission profile in varied ecotypes. The study also portrayed the actual microclimatic profile of the ambient environment and the estimates of EIP for *P. vivax* and *P. falciparum*, indicating the transmission window period which would be useful for devising appropriate vector control strategies.

## Additional files

**Additional file 1.** Month-wise mean, minimum, maximum temperature and relative humidity (RH) variations observed during the diurnal and nocturnal period in indoor and outdoor environments.

**Additional file 2.** Month-wise diurnal/nocturnal variations in temperature and relative humidity.

**Additional file 3.** Month-wise pattern of temperature (a), relative humidity (b), daily temperature range and daily relative humidity range (c), rainfall (d), man-hour density of *Anopheles stephensi* (e), malaria prevalence of the study area from 2006 to 2013 (f).

## Abbreviations

RH: relative humidity
DTR: daily temperature range
DRHR: daily relative humidity range
NVBDCP: National Vector Borne Disease Control Programme
UMS: urban malaria scheme
EIP: extrinsic incubation period
MHD: man-hour density
