# Supplementary material for: Microclimate variables of the ambient environment deliver the actual estimates of the extrinsic incubation period of Plasmodium vivax and Plasmodium falciparum: a study from a malaria-endemic urban setting, Chennai in India
Source: Malar J. 2018 May 16;17:201. doi: 10.1186/s12936-018-2342-1 (PMC5956829; doi:10.1186/s12936-018-2342-1)
Supplement: Supplementary file 1 — Additional file 1. Month-wise mean, minimum, maximum temperature and relative humidity (RH) variations observed during the diurnal and nocturnal period in indoor and outdoor environments. [file 12936_2018_2342_MOESM1_ESM.docx]

| **Month** | **Indoor/Outdoor** | **Day/Night** | **Temperature (°C)** | | **Relative humidity (%)** | |
| --- | --- | --- | --- | --- | --- | --- |
|  |  |  | **Mean (Min, Max)** | **95% CI** | **Mean (Min, Max)** | **95% CI** |
| **Nov ‘12** | **Indoor** | **Day** | 29.85 (22.91 - 36.3) | 29.8 - 29.91 | 71 (34.29 - 94.18) | 70.69 - 71.31 |
|  |  | **Night** | 28.92 (23.29 - 33.43) | 28.87 - 28.96 | 73.01 (50.51 - 93.45) | 72.75 - 73.27 |
|  | **Outdoor** | **Day** | 29.49 (21.47 - 44.7) | 29.43 - 29.55 | 79.21 (29.72 - 103.66) | 78.99 - 79.43 |
|  |  | **Night** | 27.25 (21.09 - 33.85) | 27.22 - 27.28 | 79.58 (39.68 - 103.65) | 79.37 - 79.79 |
| **Dec** | **Indoor** | **Day** | 29.16 (24.74 - 35.76) | 29.12 - 29.21 | 72.16 (46.53 - 96.44) | 71.88 - 72.45 |
|  |  | **Night** | 28.28 (25.03 - 31.78) | 28.25 - 28.25 | 73.61 (56.12 - 94.15) | 73.37 - 73.85 |
|  | **Outdoor** | **Day** | 28.7 (22.43 - 47.11) | 28.64 - 28.76 | 79.82 (43.76 - 105.14) | 79.63 - 80.02 |
|  |  | **Night** | 26.63 (22.14 - 32.81) | 26.61 - 26.65 | 80.22 (52.38 - 105.13) | 80.03 - 80.41 |
| **Jan ‘13** | **Indoor** | **Day** | 28.93 (23.58 - 35.11) | 28.88 - 28.98 | 68.73 (48.95 - 89.95) | 68.53 - 68.94 |
|  |  | **Night** | 28.24 (23.87 - 33.43) | 28.2 - 28.28 | 69.55 (48.39 - 86.72) | 69.38 - 69.72 |
|  | **Outdoor** | **Day** | 28.58 (21.19 - 46.59) | 28.52 - 28.64 | 77.71 (34.77 - 105.29) | 77.52 - 77.9 |
|  |  | **Night** | 26.48 (20.9 - 44.83) | 26.45 - 26.5 | 78.4 (50.73 - 105.29) | 78.23 - 78.57 |
| **Feb** | **Indoor** | **Day** | 29.08 (24.06 - 33.22) | 29.03 - 29.13 | 69.06 (42.55 - 92.33) | 68.8 - 69.31 |
|  |  | **Night** | 29.39 (24.26 - 34.27) | 29.34 - 29.44 | 68.98 (43.43 - 94.37) | 68.74 - 69.22 |
|  | **Outdoor** | **Day** | 28.36 (21.66 - 48.16) | 28.29 - 28.42 | 76.85 (28.88 - 98.9) | 76.64 - 77.06 |
|  |  | **Night** | 28.03 (22.43 - 48.96) | 27.97 - 28.09 | 77.06 (45.34 - 98.7) | 76.87 - 77.24 |
| **Mar** | **Indoor** | **Day** | 30.4 (25.51 - 35.44) | 30.35 - 30.45 | 70.47 (47.88 - 87.31) | 70.28 - 70.65 |
|  |  | **Night** | 31.16 (26.29 - 36.19) | 31.11 - 31.21 | 68.78 (37.85 - 91.58) | 68.55 - 69.02 |
|  | **Outdoor** | **Day** | 28.78 (23 - 46.85) | 28.73 - 28.83 | 79.02 (33.65 - 100.28) | 78.82 - 79.23 |
|  |  | **Night** | 30.64 (23.68 - 54.11) | 30.56 - 30.71 | 78.12 (32.32 - 100) | 77.9 - 78.34 |
| **Apr** | **Indoor** | **Day** | 32.67 (27.37 - 38.49) | 32.62 - 32.72 | 72.08 (50.48 - 88.6) | 71.9 - 72.26 |
|  |  | **Night** | 31.8 (27.37 - 38.71) | 31.75 - 31.85 | 74.77 (46.17 - 94.41) | 74.6 - 74.6 |
|  | **Outdoor** | **Day** | 32.56 (25.32 - 58.96) | 32.49 - 32.63 | 78.92 (34.29 - 103.95) | 78.71 - 79.13 |
|  |  | **Night** | 30.13 (25.13 - 42.52) | 30.1 - 30.16 | 80.69 (39.34 - 102.07) | 80.5 - 80.89 |
| **May** | **Indoor** | **Day** | 33.09 (27.96 - 42.16) | 33.04 - 33.14 | 68.97 (28.36 - 87.96) | 68.67 - 69.27 |
|  |  | **Night** | 32.89 (28.16 - 40.3) | 32.84 - 32.94 | 70.02 (27.34 - 91.65) | 69.72 - 70.31 |
|  | **Outdoor** | **Day** | 32.65 (26 - 61.38) | 32.58 - 32.72 | 77.87 (23.03 - 105.25) | 77.67 - 78.08 |
|  |  | **Night** | 31.9 (25.9 - 56.84) | 31.85 - 31.95 | 77.42 (18.51 - 103.89) | 77.2 - 77.64 |
| **Jun** | **Indoor** | **Day** | 33.36 (26.88 - 41.81) | 33.29 - 33.43 | 54.8 (30.04 - 90.71) | 54.46 - 55.14 |
|  |  | **Night** | 32.15 (26.88 - 37.17) | 32.1 - 32.2 | 63.33 (35.63 - 89.24) | 63.02 - 63.65 |
|  | **Outdoor** | **Day** | 32.79 (25.03 - 60.16) | 32.72 - 32.87 | 71.55 (22.6 - 105.41) | 71.22 - 71.87 |
|  |  | **Night** | 30.52 (24.35 - 59.3) | 30.48 - 30.56 | 74.84 (42.29 - 105.29) | 74.56 - 75.12 |
| **Jul** | **Indoor** | **Day** | 30.83 (25.81 - 39.28) | 30.77 - 30.89 | 67.21 (33.8 - 94.22) | 66.87 - 67.56 |
|  |  | **Night** | 30.25 (25.81 - 35.44) | 30.2 - 30.3 | 72.93 (37.88 - 93.2) | 72.65 - 73.21 |
|  | **Outdoor** | **Day** | 30.83 (23.87 - 57.16) | 30.76 - 30.9 | 79.58 (27.88 - 104.27) | 79.34 - 79.82 |
|  |  | **Night** | 28.65 (23.58 - 37.06) | 28.61 - 28.68 | 81.24 (40.36 - 103.83) | 81.03 - 81.45 |
| **Aug** | **Indoor** | **Day** | 30.67 (24.64 - 37.6) | 30.61 - 30.74 | 69.88 (34.92 - 92.28) | 69.55 - 70.22 |
|  |  | **Night** | 30.12 (24.64 - 35.22) | 30.06 - 30.17 | 75.16 (41.95 - 98.89) | 74.93 - 75.4 |
|  | **Outdoor** | **Day** | 31.01 (23.48 - 57.97) | 30.93 - 31.09 | 78.94 (38.06 - 104.34) | 78.71 - 79.16 |
|  |  | **Night** | 28.61 (23.48 - 37.82) | 28.58 - 28.64 | 81.29 (44.6 - 103.65) | 81.1 - 81.47 |
| **Sep** | **Indoor** | **Day** | 30.76 (24.84 - 38.16) | 30.7 - 30.82 | 72.05 (37.14 - 96.69) | 71.7 - 72.4 |
|  |  | **Night** | 30.02 (24.84 - 35.22) | 29.97 - 30.07 | 76.94 (47.99 - 96.5) | 76.7 - 77.19 |
|  | **Outdoor** | **Day** | 30.79 (23.48 - 60.5) | 30.71 - 30.86 | 81.92 (36.1 - 104.32) | 81.71 - 82.14 |
|  |  | **Night** | 28.53 (23.29 - 35.54) | 28.5 - 28.56 | 84.51 (53.96 - 103.67) | 84.33 - 84.68 |
| **Oct** | **Indoor** | **Day** | 30.67 (24.55 - 37.71) | 30.61 - 30.73 | 72.59 (40.43 - 95.4) | 72.27 - 72.91 |
|  |  | **Night** | 30.12 (25.13 - 34.8) | 30.07 - 30.17 | 75.9 (50.72 - 95.37) | 75.67 - 76.13 |
|  | **Outdoor** | **Day** | 30.46 (23.48 - 63.02) | 30.38 - 30.54 | 81.52 (39.44 - 101.81) | 81.31 - 81.72 |
|  |  | **Night** | 28.22 (23.58 - 34.59) | 28.19 - 28.25 | 83.26 (55.87 - 100.42) | 83.08 - 83.43 |

**Additional file 1:** Month-wise mean, minimum, maximum temperature and relative humidity (RH) variations observed during diurnal and nocturnal period in indoor and outdoor environments
