# Supplementary figures and images for: Microclimate variables of the ambient environment deliver the actual estimates of the extrinsic incubation period of Plasmodium vivax and Plasmodium falciparum: a study from a malaria-endemic urban setting, Chennai in India
Source: Malar J. 2018 May 16;17:201. doi: 10.1186/s12936-018-2342-1 (PMC5956829; doi:10.1186/s12936-018-2342-1)

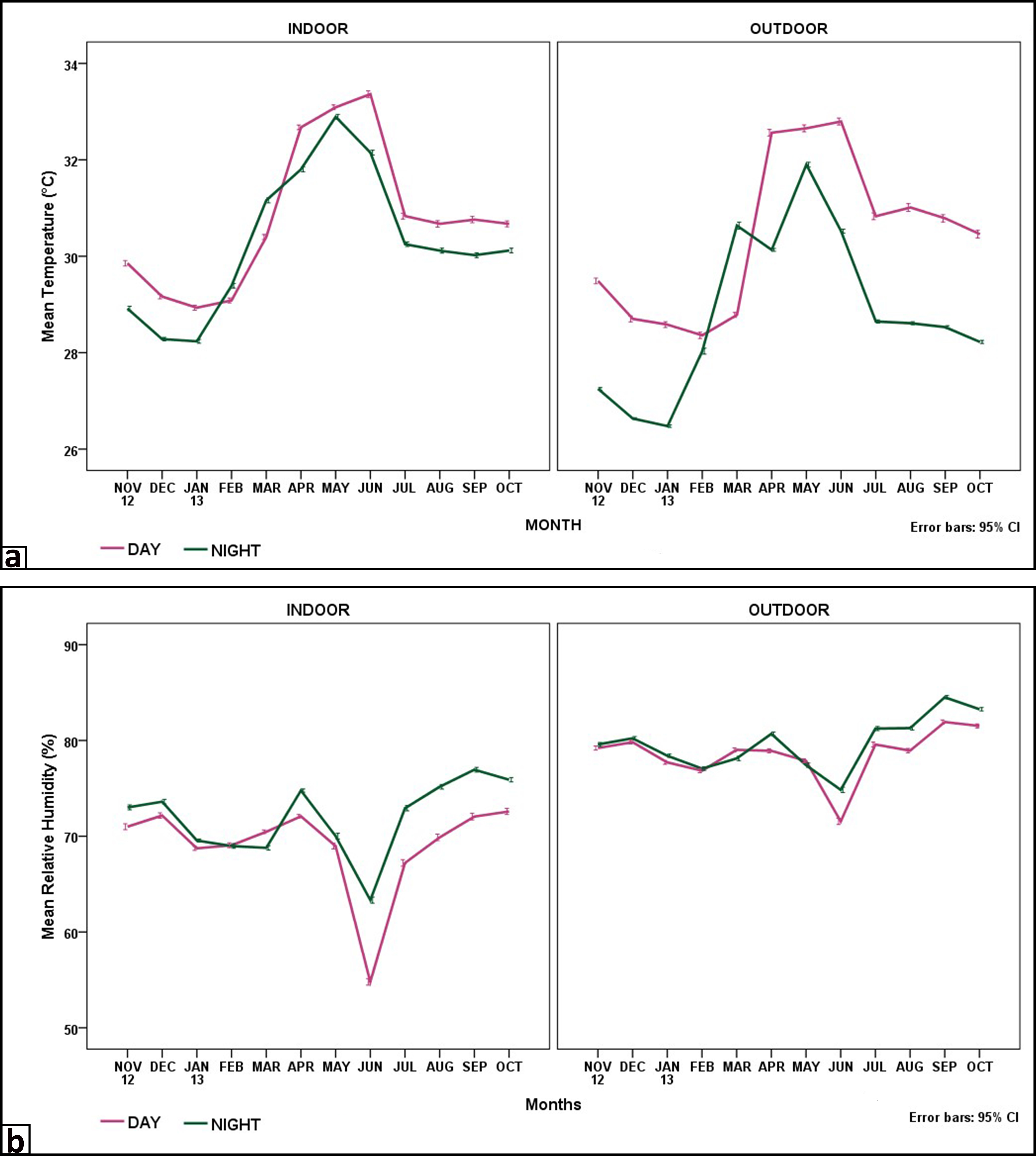

Supplement: Supplementary file 2 — Additional file 2. Month-wise diurnal/nocturnal variations in temperature and relative humidity. [file 12936_2018_2342_MOESM2_ESM.jpg]

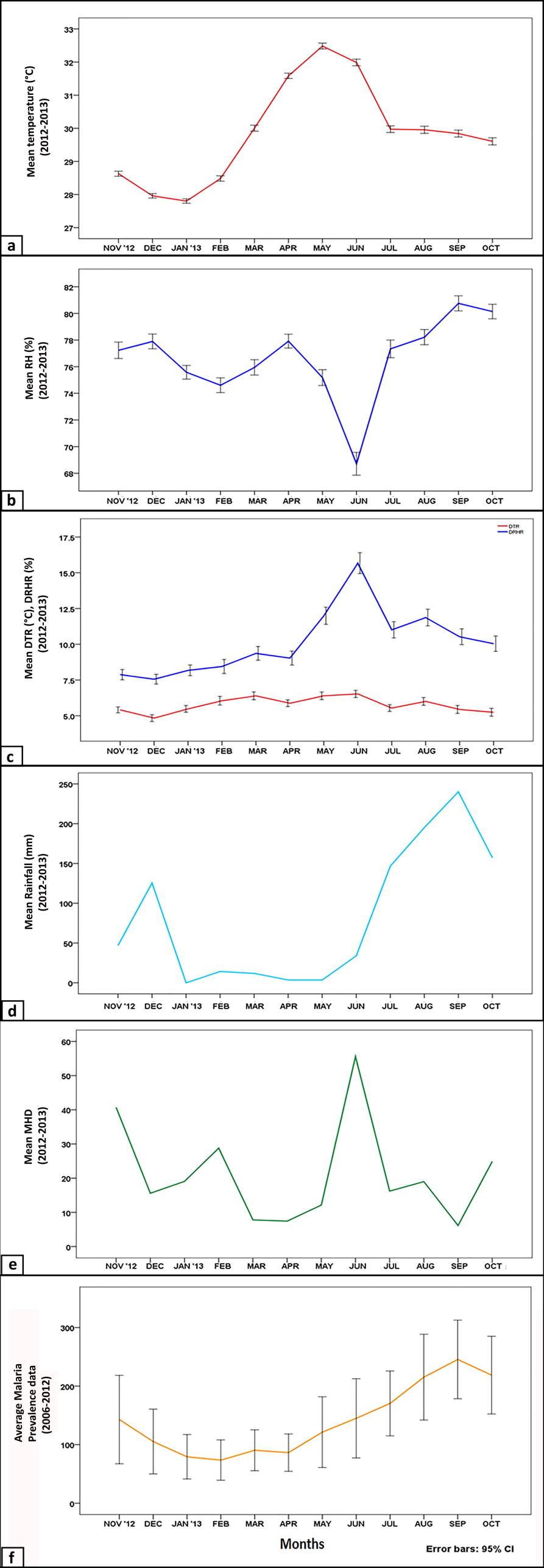

Supplement: Supplementary file 3 — Additional file 3. Month-wise pattern of temperature (a), relative humidity (b), daily temperature range and daily relative humidity range (c), rainfall (d), man-hour density of Anopheles stephensi (e), malaria prevalence of the study area from 2006 to 2013 (f). [file 12936_2018_2342_MOESM3_ESM.jpg]
